# Diagnostic Accuracy of Serum Anti-phospholipase A2 Receptor (PLA2R) Antibody Assays for Membranous Nephropathy in Patients With Nephrotic Syndrome: A Systematic Review

**DOI:** 10.7759/cureus.97957

**Published:** 2025-11-27

**Authors:** Sally Hamad, Haneen Maan Alkaales, Rawshan Nassrullah, Rawan Nazar Qasim, Sarah Nezar Qasim, Abdulrahman Al-Mohammed

**Affiliations:** 1 General Medicine, Peterborough City Hospital, Peterborough, GBR; 2 General Medicine, Furness General Hospital, University Hospitals of Morecambe Bay NHS Foundation Trust, Barrow-in-Furness, GBR; 3 Emergency Medicine, University Hospital of North Tees, Stockton-on-Tees, GBR; 4 Psychiatry, Ibn Rushd Psychiatric Hospital, Baghdad, IRQ; 5 Otolaryngology, Al-Karama Teaching Hospital, Baghdad, IRQ; 6 Oncology and Radiation Therapy, Mordovia State University, Saransk, RUS

**Keywords:** anti-phospholipase a2 receptor (pla2r), diagnostic test accuracy, kidney biopsy, membranous nephropathy, nephrotic syndrome

## Abstract

Primary membranous nephropathy (MN) is a common cause of nephrotic syndrome in adults, and circulating anti-phospholipase A2 receptor (PLA2R) antibodies have emerged as a key diagnostic biomarker. Following the Preferred Reporting Items for Systematic Reviews and Meta-Analyses (PRISMA) 2020 guidelines, we conducted a systematic review of eight studies evaluating serum PLA2R antibody testing against kidney biopsy, with sample sizes ranging from 117 to 670 participants. Extracted data included assay type, cutoff values, sensitivity, specificity, and area under the curve (AUC), where available. Across 2,600+ patients, reported sensitivities ranged from 60% to 80.75%, while specificity was generally ≥90%, and five studies reported AUCs between 0.83 and 0.935, with a weighted mean AUC of 0.877. Lower cutoffs (~2-3 RU/ml) increased sensitivity at the cost of slightly reduced specificity. In contrast, higher cutoffs (>20 RU/ml) improved specificity at the cost of lower sensitivity, and combined ELISA and confirmatory indirect immunofluorescence further increased specificity to 100% but decreased sensitivity. These findings indicate that serum PLA2R antibody testing is a reliable rule-in diagnostic tool for MN, although negative results cannot reliably exclude the disease. Future large-scale prospective studies with standardized cutoffs and complete 2 × 2 contingency data are needed to enable formal meta-analytic pooling and optimize diagnostic thresholds and cutoff selection for clinical practice.

## Introduction and background

Membranous nephropathy (MN) is one of the most common causes of adult-onset nephrotic syndrome, accounting for up to one-third of biopsy-proven cases worldwide. It is characterized histologically by diffuse thickening of the glomerular basement membrane and subepithelial immune complex deposits, leading to heavy proteinuria, hypoalbuminemia, and edema. MN can be either primary (idiopathic) or secondary to systemic conditions such as autoimmune diseases, infections, malignancies, or drug exposure. Differentiating between primary and secondary forms is essential because therapeutic strategies and prognoses differ significantly [1].

Traditionally, diagnosis has relied on a kidney biopsy, which remains the gold standard for confirming MN. However, biopsy is invasive, may be contraindicated in some patients, and carries risks such as bleeding and sampling error [2,3]. These limitations have driven the search for reliable noninvasive biomarkers to support, or, in select cases, replace biopsy in diagnosis and disease monitoring.

The identification of the M-type phospholipase A2 receptor (PLA2R) as the major autoantigen in primary MN has transformed the diagnostic approach to this disease [4]. Circulating anti-PLA2R antibodies are present in most patients with primary MN and are rarely detected in secondary forms or other glomerulopathies, making them a specific biomarker [5]. The development of serological assays for anti-PLA2R antibodies has enabled noninvasive diagnosis and monitoring of disease activity. Among these, enzyme-linked immunosorbent assay (ELISA) and indirect immunofluorescence (IIF) are the most widely used platforms, offering a noninvasive diagnostic tool [6].

Multiple diagnostic studies have assessed the clinical performance of these assays in patients presenting with nephrotic syndrome. Reported sensitivity and specificity values have varied across studies, likely reflecting differences in assay methodology, cutoff selection, patient populations, and timing of sample collection [7]. Despite widespread clinical adoption, uncertainty persists regarding optimal diagnostic thresholds and the influence of patient- and disease-related factors on test performance. The lack of harmonized testing standards continues to challenge consistent clinical interpretation and integration of serological results into diagnostic decision-making.

This systematic review aims to consolidate current evidence regarding the diagnostic performance of serum anti-PLA2R antibody assays for detecting primary MN among patients presenting with nephrotic syndrome. By integrating data from recent diagnostic accuracy studies, this review seeks to clarify reported sensitivity, specificity, positive and negative predictive values (PPV and NPV), and area under the curve (AUC) values across different cutoff points, identify sources of heterogeneity, and evaluate whether serological testing can reliably complement or substitute for kidney biopsy. Through this review, we aim to provide clinicians and researchers with an up-to-date, evidence-based understanding of the accuracy, clinical utility, and limitations of PLA2R antibody testing in the diagnostic pathway of MN.

## Review

Methods

Study Design

This systematic review was designed to evaluate the diagnostic accuracy of serum PLA2R antibodies for detecting MN among patients presenting with nephrotic syndrome. The study followed the Preferred Reporting Items for Systematic Reviews and Meta-Analyses of Diagnostic Test Accuracy Studies (PRISMA-DTA) guidelines [8]. The review was prospectively registered in PROSPERO with the registration number CRD420251161339.

Eligibility Criteria

The inclusion criteria for this systematic review were defined according to the PIRD framework (population, index test, reference standard, diagnosis) to ensure the selection of studies directly relevant to the diagnostic accuracy of serum PLA2R antibodies in MN. Eligible studies included adult participants aged 18 years and older who presented with nephrotic syndrome and underwent evaluation for MN (population). Studies were required to assess serum PLA2R antibody assays, irrespective of assay type (e.g., ELISA, IIF, or other validated laboratory techniques; index test), and to use renal biopsy as the reference standard (reference standard) for confirming MN diagnosis (diagnosis). Only original research reporting sufficient data to determine diagnostic accuracy parameters, including sensitivity and specificity, or data suitable for constructing 2 × 2 contingency tables, was considered. Both cross-sectional and cohort studies, as well as case-control designs involving at least 10 participants, were eligible for inclusion.

Exclusion criteria encompassed studies that focused solely on disease prognosis or treatment response without a diagnostic component. Research lacking a histopathological reference standard or insufficient data to evaluate diagnostic performance was excluded. Conference abstracts, editorials, commentaries, and unpublished studies without peer-reviewed results were also omitted. We excluded studies involving individuals under 18 years of age and those involving animals. Only articles published in the English language between May 2015 and May 2025 were included in this review.

Search Strategy

A comprehensive literature search was conducted to identify studies evaluating the diagnostic accuracy of serum PLA2R antibodies in patients presenting with nephrotic syndrome. Searches were conducted in PubMed, MEDLINE, Web of Science, Scopus, Google Scholar, and the Cochrane Library databases from May 2015 to May 2025. Only studies published in the English language were considered eligible for inclusion.

The search strategy was developed using a combination of Medical Subject Headings (MeSH) and free-text terms associated with MN, PLA2R antibodies, and diagnostic accuracy. Boolean operators “AND” and “OR” were employed to combine search terms and enhance sensitivity and specificity (Table 1).

**Table 1:** Search strategy

Database	Search strategy
PubMed/MEDLINE	{[Glomerulonephritis, Membranous (MeSH) OR Membranous Glomerulonephritis (tiab) OR Membranous Glomerulopathy (tiab) OR Membranous Nephropathy (tiab)] AND [Receptors, Phospholipase A2 (MeSH) OR PLA(2) Receptor OR Phospholipase A2 Receptor OR Anti-Phospholipase A2 Receptor OR M-type Phospholipase A2 Receptor OR anti-PLA2R OR aPLA2R OR PLA2R antibody] AND [Sensitivity and Specificity (MeSH) OR Sensitiv* OR Specific* OR Diagnostic Accuracy (tiab) OR Diagnos*]}.
Web of Science	{TS=("membranous nephropathy" OR "membranous glomerulonephritis" OR "membranous glomerulopathy") AND TS=("phospholipase A2 receptor" OR "anti-PLA2R" OR "PLA2R antibody") AND TS=("diagnostic accuracy" OR "sensitivity" OR "specificity" OR "diagnostic performance")}.
Scopus	{TITLE-ABS-KEY(("membranous nephropathy" OR "membranous glomerulonephritis" OR "membranous glomerulopathy") AND ("phospholipase A2 receptor" OR "anti-PLA2R" OR "PLA2R antibody") AND ("diagnostic accuracy" OR "sensitivity" OR "specificity" OR "predictive value"))}.
Google Scholar	{"membranous nephropathy" AND "phospholipase A2 receptor" AND ("diagnostic accuracy" OR "sensitivity" OR "specificity")}. Only the first 200 most relevant results were screened to ensure specificity.
Cochrane Library	{[Membranous Glomerulonephritis OR Membranous Glomerulopathy OR Membranous Nephropathy OR MeSH descriptor: (Glomerulonephritis, Membranous) explode all trees]} AND {[PLA(2) Receptor OR Phospholipase A2 Receptor OR M-type Phospholipase A2 Receptor OR anti-PLA2R OR PLA2R OR MeSH descriptor: (Receptors, Phospholipase A2) explode all trees]}.

Screening and Study Selection

Two authors conducted a rigorous two-phase screening process to ensure eligible studies were included. In the first phase, titles and abstracts were screened to exclude records that were clearly irrelevant based on the predefined inclusion and exclusion criteria. In the second phase, the full texts of potentially relevant studies were retrieved and assessed for eligibility. Studies were included if they evaluated serum PLA2R antibody assays as a diagnostic test in patients with nephrotic syndrome, used renal biopsy as the reference standard, and reported or allowed calculation of diagnostic accuracy measures such as sensitivity and specificity. Any disagreements between the two reviewers during either phase of screening were resolved through discussion, and when necessary, a third reviewer was consulted to achieve consensus. The final selection of studies was based on mutual agreement following this adjudication process.

Quality Assessment

The methodological quality and risk of bias for included studies were assessed using the Quality Assessment of Diagnostic Accuracy Studies (QUADAS-2) tool [9]. Each study was evaluated across four domains: patient selection, index test, reference standard, and flow and timing. Each domain included flagging questions with responses of "Yes," "No," or "Unclear." The overall risk of bias was assessed as high when answered "No" to any question, low when responded "Yes" to all inquiries, and unclear when replied "Unclear" to all questions or combined with any "Yes."

Data Synthesis and Analysis

Data synthesis and analysis were conducted using a structured, descriptive approach. Extracted data were organized into summary tables detailing key characteristics of the included studies, including author information, year, country, sample size, assay method, cutoff values, and reported diagnostic parameters. A narrative synthesis was then employed to summarize and interpret the diagnostic performance of serum PLA2R antibody assays, integrating the findings across various assay types, cutoff thresholds, and patient populations. This approach allowed for the identification of key trends, methodological variations, and population-specific factors influencing diagnostic accuracy.

Results

Study Selection

**Figure 1:**
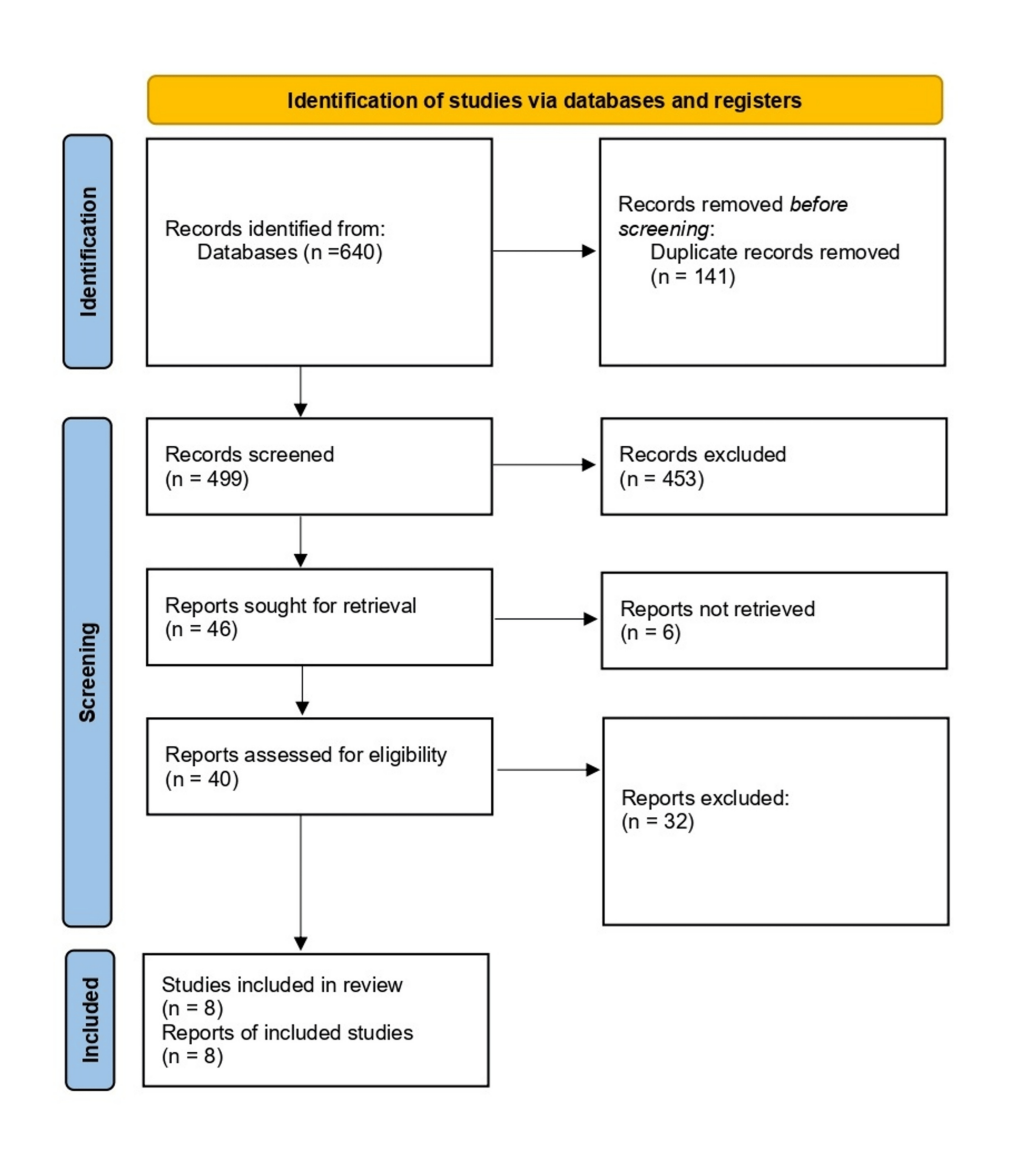
PRISMA 2020 flow diagram outlining the identification, screening, and selection of articles for this systematic review PRISMA: Preferred Reporting Items for Systematic Reviews and Meta-Analysis [8]

Upon completing the comprehensive database search, 640 records were identified across PubMed, MEDLINE, Web of Science, Scopus, Google Scholar, and the Cochrane Library. After removing 141 duplicate records, 499 studies remained for screening. Titles and abstracts were reviewed against the predefined inclusion and exclusion criteria, resulting in the exclusion of 453 records that did not meet eligibility requirements. Forty-six reports were sought for retrieval, of which six could not be obtained. A total of 40 full-text articles were assessed for eligibility, and 32 studies were excluded for not meeting the diagnostic accuracy criteria or lacking biopsy-confirmed MN as a reference standard. Ultimately, eight studies [10-17] met the inclusion criteria and were included in this systematic review. The detailed process of study identification, screening, and inclusion is illustrated in the PRISMA 2020 flow diagram (Figure 1). Duplicate records were removed using EndNote (Clarivate, Philadelphia, PA, USA) [18].

Methodological Quality Assessment and Risk of Bias

**Figure 2:**
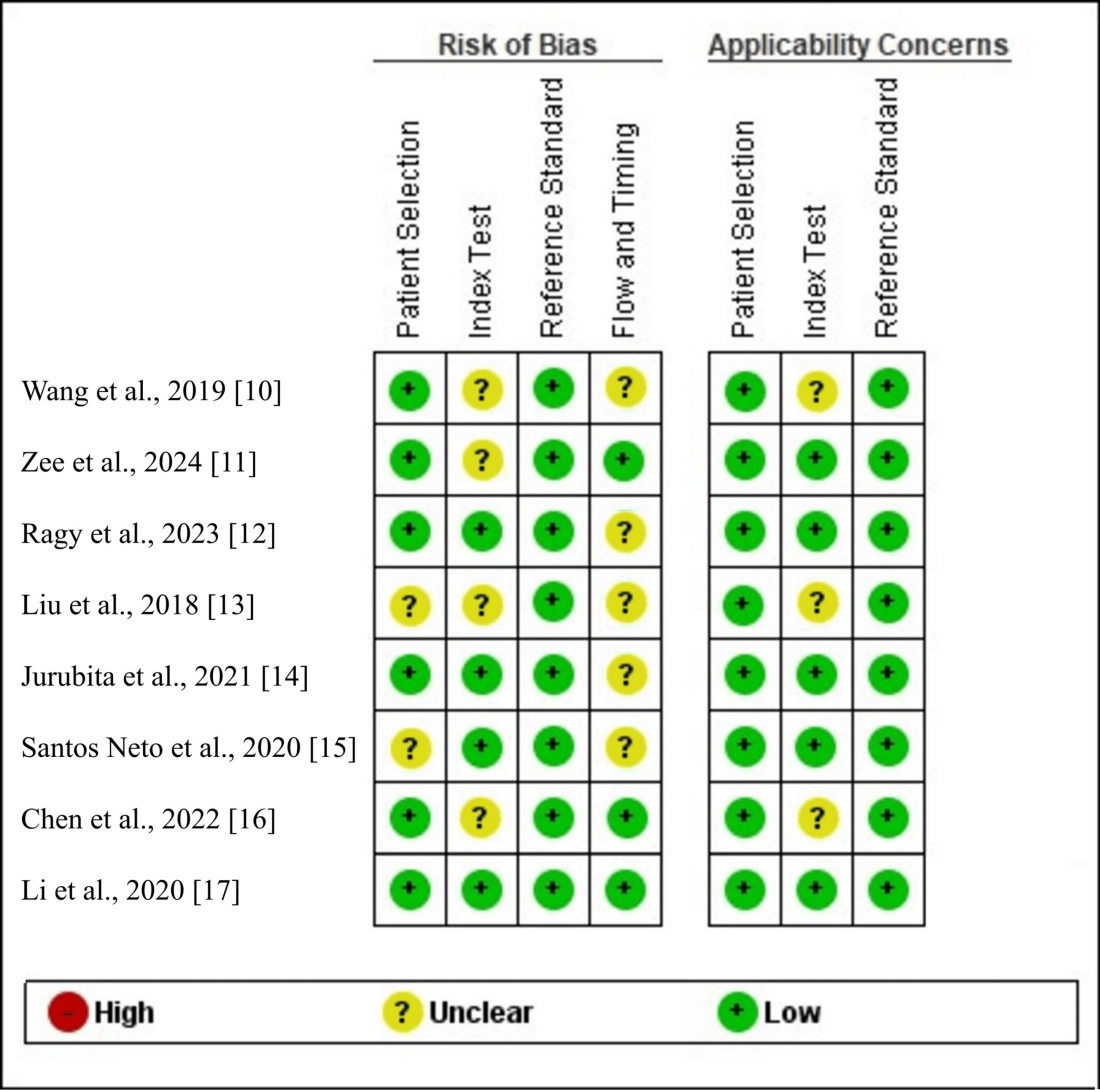
Risk of bias assessment based on the QUADAS-2 evaluation The figure was created by the authors using RevMan software version 5.4 [19]. QUADAS-2: Quality Assessment of Diagnostic Accuracy Studies [9]

Based on the QUADAS-2 evaluation, the overall methodological quality of the included studies was acceptable but limited by incomplete reporting. Most studies enrolled appropriate patient populations, indicating low risk in the patient selection domain, although only a few explicitly described consecutive sampling. The index test domain showed unclear risk across multiple studies due to insufficient blinding descriptions and variability in cutoff thresholds. All studies appropriately used renal biopsy as the reference standard, resulting in low concern for the reference standard domain. A frequent source of bias was the flow and timing domain, as only three studies reported the interval between antibody testing and biopsy. The summarized quality assessment and risk of bias are shown in Figure 2. Quality assessment and risk of bias were generated using the RevMan software version 5.4 (Cochrane Collaboration, London, UK) [19].

Study Characteristics

As shown in Table 2, eight studies published between 2018 and 2024 that evaluated the diagnostic accuracy of serum anti-PLA2R antibodies for diagnosing MN in patients with nephrotic syndrome were included in this systematic review [10-17]. The studies were conducted in China [10,13,16,17], the United Kingdom [12], the United States [11], Romania [14], and Brazil [15]. All studies used ELISA for PLA2R detection, while Zee et al. [11] additionally incorporated IIF for borderline results (2-20 RU/mL). Four studies specified the Euroimmun assay, and four did not mention the manufacturer. Cutoff thresholds varied widely, between 2 and 20 RU/mL, with the most common being ≥20 RU/mL (three studies) [12,14,15]. Reported sensitivities ranged from 60.0% to 80.75%, while specificities were generally high (≥90%) in all studies. PPV ranged between 80% and 100%, and NPV ranged between 67.9% and 95.1%. The AUC across five studies ranged from 0.83 to 0.935, indicating good overall diagnostic performance. Population-specific differences were noted. Wang et al. [10] reported high specificity (95.1%) and good sensitivity (80.0%) in diabetic kidney disease. Jurubita et al. [14] observed improved sensitivity in younger patients with preserved renal function. Ragy et al. [12] reported that higher antibody titers were associated with more severe disease. Li et al. [17] found antibody levels correlated with proteinuria and kidney function, while Zee et al. [11] showed that combining ELISA with IIF yielded 100% specificity and PPV but decreased sensitivity.

**Table 2:** Characteristics of the included studies ELISA: enzyme-linked immunosorbent assay, IIF: indirect immunofluorescence, PPV: positive predictive value, NPV: negative predictive value, AUC: area under the curve

Author, year	Country	Sample size	Assay method	Cutoff value(s) tested (RU/ml)	Sensitivity (%)	Specificity (%)	PPV (%)	NPV (%)	AUC
Wang et al., 2019 [10]	China	227	ELISA (manufacturer not specified)	≥2.71	80.0	95.1	80.0	95.1	0.87
Zee et al., 2024 [11]	United States	468	ELISA + IIF (manufacturer not specified)	≥2	60.0	100.0	100.0	92.0	Not reported
Ragy et al., 2023 [12]	United Kingdom	187	ELISA (Euroimmun)	≥20	75.5	97.8	97.3	79.8	Not reported
Liu et al., 2018 [13]	China	141	ELISA (manufacturer not specified)	≥2.6	78.9	91.7	86.5	86.5	0.879
Jurubita et al., 2021 [14]	Romania	203	ELISA (Euroimmun)	≥20	64.0	94.0	91.0	75.0	0.83
Santos Neto et al., 2020 [15]	Brazil	117	ELISA (Euroimmun)	≥20	60.5	94.7	92.9	67.9	Not reported
Chen et al., 2022 [16]	China	589	ELISA (manufacturer not specified)	≥3.8	71.0	90.0	88.0	75.0	0.83
Li et al., 2020 [17]	China	670	ELISA (Euroimmun)	≥7.45	80.75	97.97	98.05	80.11	0.935

Discussion

This systematic review consolidates current evidence on the diagnostic accuracy of serum anti-PLA2R antibodies in distinguishing primary MN from other causes of nephrotic syndrome in adults. Across eight studies encompassing more than 2,600 participants, serum PLA2R testing demonstrated consistently high specificity (≥90%) but variable sensitivity (60-80.75%), supporting its role as a highly specific but moderately sensitive marker for MN. The diagnostic performance, as reflected in an average AUC of 0.877 across studies, confirms its strong overall discriminative ability. These findings align with prior research, which has established anti-PLA2R antibodies as a reliable serological hallmark of primary MN and a practical complement to renal biopsy in appropriate clinical contexts.

Diagnostic Performance and Clinical Implications

The high specificity observed across all included studies underscores the value of anti-PLA2R testing as a rule-in diagnostic tool. Positive antibody detection in a patient with nephrotic-range proteinuria and compatible clinical features can obviate the immediate need for invasive biopsy in selected cases, a concept increasingly reflected in recent clinical practice trends and acknowledged in clinical guidelines [20]. In contrast, the variability in sensitivity indicates that a negative test cannot reliably exclude MN, especially in early or atypical presentations. Factors influencing sensitivity include disease stage, antibody kinetics, age, and assay thresholds.

Effect of Cutoff Thresholds and Assay Methodology

Heterogeneity in assay cutoffs was a significant determinant of diagnostic variability. Lower thresholds (approximately 2-3 RU/mL) tended to enhance sensitivity at the expense of specificity, whereas higher cutoffs (>20 RU/mL) achieved near-perfect specificity at the expense of reduced sensitivity. For example, Santos Neto et al. [15] and Jurubita et al. [14], both using a 20 RU/mL threshold, reported specificities above 94% but sensitivities around 60-65%. Conversely, studies employing lower cutoffs, such as Liu et al. [13] and Wang et al. [10], achieved sensitivities around 80% while maintaining acceptable specificity. These findings suggest that optimizing cutoff selection according to clinical context, screening versus confirmatory testing, may maximize diagnostic yield.

Regarding assay types, the ELISA was used in all studies and remains the standard due to its quantifiable nature and ease of standardization. However, Zee et al. [11] demonstrated that combining ELISA with confirmatory IIF further improved specificity to 100%, reinforcing the benefit of multimodal testing in ambiguous cases. Future diagnostic protocols may integrate both assays, reserving IIF for borderline ELISA results to improve confidence in positive or equivocal findings.

Population and Disease-Related Influences

Population-specific trends also emerged. Li et al. [17] observed strong correlations between antibody titers, proteinuria, and renal function, supporting the biological plausibility that circulating PLA2R levels mirror disease activity. Jurubita et al. [14] reported improved sensitivity among younger patients with preserved renal function, possibly reflecting more robust antibody production in earlier disease stages. Conversely, reduced sensitivity in older or advanced cases may stem from antibody depletion caused by immune complex deposition or by immunosuppressive therapy prior to testing.

Comparison With Previous Literature

The findings of this review are consistent with earlier meta-analyses, which reported pooled sensitivities of 63-67% and specificities exceeding 97% for anti-PLA2R testing [7]. The present synthesis reinforces these observations while emphasizing the influence of methodological variability on performance. Additionally, the review underscores the need for harmonized reporting of cutoff values, reference ranges, and test calibration to reduce inter-study heterogeneity.

Strengths and Limitations

A key strength of this review lies in its adherence to PRISMA-DTA [8] standards and inclusion of recent, biopsy-confirmed diagnostic studies. The diversity of populations and assay platforms enhances generalizability. However, several limitations must be acknowledged. First, incomplete reporting in primary studies limited formal meta-analytic pooling and precluded subgroup analyses. Second, variations in assay manufacturers, cutoff definitions, and timing between serum sampling and biopsy contributed to heterogeneity. Third, some studies lacked blinding of assessors, potentially biasing the interpretation of the index test. Lastly, excluding non-English literature may have introduced language bias.

Future Directions

Future research should prioritize prospective multicenter studies with standardized assay calibration, uniform cutoff definitions, and complete 2 × 2 contingency data to facilitate meta-analytic synthesis. Combining serological testing with immunohistochemical staining of renal biopsies for PLA2R and other target antigens could further refine diagnostic stratification. Moreover, longitudinal studies evaluating antibody dynamics in relation to treatment response and relapse risk will enhance the clinical utility of PLA2R quantification beyond diagnosis.

## Conclusions

Serum anti-PLA2R antibody testing demonstrates excellent specificity and good overall diagnostic accuracy for primary MN, making it a valuable noninvasive tool for confirming diagnosis in patients with nephrotic syndrome. However, variable sensitivity and assay heterogeneity limit its use as a standalone exclusion test. Integrating serological results with clinical assessment and, when necessary, renal histopathology remains the most reliable diagnostic approach. Continued efforts to standardize assays and optimize thresholds are essential to maximize the test’s clinical applicability and reproducibility.
